# Pregnancy hormones increase cardiac capillary density via the PGC-1α/ERRα/VEGF pathway in cardiomyocytes

**DOI:** 10.3389/fcvm.2025.1688831

**Published:** 2025-10-14

**Authors:** Michael Hesse, Daniel Korzus, Kristina Thaben, Nicole Wagner, Süleyman Ergün, Zoltan Arany, Bernd K. Fleischmann

**Affiliations:** ^1^ Institute of Physiology I, Medical Faculty, University of Bonn, Bonn, Germany; ^2^ Institute of Anatomy, Julius-Maximilians-University Würzburg, Würzburg, Germany; ^3^ Perelman School of Medicine, University of Pennsylvania, Philadelphia, PA, United States

**Keywords:** capillaries, hormones, angiogenesis, heart, pregnancy

## Abstract

**Background:**

Pregnancy significantly affects the maternal cardiovascular system, with physiological adaptations characterized by cardiac hypertrophy and increased capillarization. However, the molecular mechanisms underlying these adaptations remain incompletely understood. Therefore, we analyzed them in mouse hearts at different stages of pregnancy and after hormone treatment.

**Methods:**

We analyzed cell proliferation, capillary density, hypertrophy, and gene expression using immunostaining and quantitative RT-PCR to evaluate differential gene expression in mouse hearts at different stages during pregnancy and after treatment with combinations of progesterone and estrogen for up to 14 days.

**Results:**

We found that the number of proliferating cells in the hearts of pregnant mice began to increase at gestational day 3 (GD3), peaked at GD14—mainly in fibroblasts and endothelial cells (ECs), but not in cardiomyocytes (CMs)—and decreased immediately after delivery. EC proliferation was indicative of angiogenesis, as evidenced by increased capillary density. After hormone treatment, capillary density increased in the hearts of both female and male mice, without prominent CM hypertrophy and independently of nuclear hormone receptors. The proportion of proliferating cardiac cells and ECs was significantly increased after 14 days of treatment. Mechanistically, we identified activation of the PGC-1α/ERRα signaling pathway and upregulation of its downstream target VEGF-A. Using a CM-specific PGC-1α knockout mouse line, we demonstrated that the pregnancy hormone-induced angiogenesis is induced via PGC-1α signaling in CMs by secretion of VEGF.

**Conclusions:**

Our data indicated a direct effect of pregnancy hormones on cardiac capillarization, rather than indirect effects through CM hypertrophy, and demonstrate that capillary expansion is not sufficient to drive physiological hypertrophy. Pregnancy hormones directly act on CMs via the PGC-1α/ERRα signaling pathway and VEGF secretion, positioning CMs as a key source of angiogenic factors that promote endothelial cell proliferation and enhance capillary density in the heart.

## 1 Introduction

Pregnancy is characterized by complex adaptive processes that affect various organ systems, including the central nervous (1) and cardiovascular system (2). Investigating the mechanisms underlying adaptive and maladaptive processes in the heart, such as hypertrophy in response to exercise, pregnancy, and hypertension, may help identify new cardioprotective pathways (3). Physiological hypertrophy occurs during postnatal heart growth, physical activity, or pregnancy (4). During pregnancy, the maternal heart responds to increased cardiac output and blood volume with eccentric hypertrophy (5). A characteristic of this physiological hypertrophy is normal or improved contractility of the cardiac muscle (6). In addition to a reversible hypertrophic growth of cardiomyocytes (CMs) (2, 7, 8), increased vascularization of the heart has also been observed (9, 10). This contrasts with pathological hypertrophy, in which chronic pressure or volume stress can result in a severe cardiovascular disease associated with increased morbidity and mortality (4). Pathological cardiac hypertrophy is characterized by structural changes, such as interstitial fibrosis and reduced capillarization of the myocardium, leading to CM death and, in the long term, impaired heart function and heart failure (11). In contrast, enhanced cardiac angiogenesis, as seen during pregnancy, may represent a potential therapeutic strategy to increase vascular density in myocardial tissue during ischemic disease and cardiac hypertrophy. However, the mechanisms underlying this physiological adaptation of the maternal heart and the potential role of pregnancy hormones are not yet fully understood. Estrogen (E2) and progesterone (P4) signal primarily through nuclear receptors, ERα, ERβ, PR-A, and PR-B (12). In the mouse heart, both ER nuclear receptors are expressed (13, 14). Studies using ERα and ERβ knockout mice have shown that E2 regulates VEGF gene induction via nuclear receptors. The deletion of ERα reduces the capillary density in the mouse heart (15), whereas the deletion of ERβ in female mice leads to increased cardiac hypertrophy after transverse aortic constriction, suggesting a protective role for ERβ (16). In addition, ERα activation has been shown to decrease hypertrophy in a model of transverse aortic constriction (17).

Cardioprotective effects of P4 have also been demonstrated in several animal models. P4 treatment enhances stroke volume and cardiac function after traumatic hemorrhagic shock (18) and reduces the infarct area in female rats by attenuating the inflammatory response (19). However, it needs to be clarified which receptors P4 uses to exert its effect, as the nuclear receptors PR-A and PR-B are not expressed in CMs (20). Moreover, peroxisome proliferator-activated receptor gamma coactivator 1-alpha (PGC-1α) has been demonstrated to mediate angiogenesis in skeletal muscle and the heart (21, 22). CM-specific knockout of PGC-1α leads to reduced VEGF expression in the hearts of female mice after pregnancy, resulting in a reduced cardiac capillary density (21).

In this study, we used pregnancy as a model to explore cardiovascular adaptation mechanisms. We show that treating female and male mice with a combination of E2 and P4 increases cardiac capillary density by inducing endothelial cell (EC) proliferation. Furthermore, using a CM-specific PGC-1α^−/−^ mouse line, we show that angiogenesis triggered by pregnancy hormones is induced by PGC-1α signaling and the secretion of VEGF from cardiomyocytes.

## 2 Materials and methods

### 2.1 Animal experimental work

For experiments with non-transgenic mice, 6–8 weeks old mice of the outbred strain CD-1 were used. Non-pregnant (NP) female mice were used as controls. For the experiments with pregnant mice, matings were set up, and females were examined daily for the formation of a vaginal plug. The day on which a plug was detected was defined as pregnancy day 0, whereas the day on which birth occurred was defined as postpartum 0 (P0). Mice without a plug were excluded from the study.

P4 receptor knockout mice were kindly provided by John. P. Lydon and Francesco J. DeMayo from Baylor College of Medicine, Houston, TX, USA. ERα^−/−^ mouse lines were obtained from The Jackson Laboratory (Stock #026176). The CM-specific PGC-1α^−/−^ mouse line was generated by crossing a transgenic mouse line expressing Cre under control of the αMHC promoter with a floxed PGC-1α mouse line. For experiments with transgenic mice, 3–5-month-old animals were used.

### 2.2 Puncture of the retrobulbar venous plexus and blood plasma collection

Mice were anesthetized with isoflurane until the respiratory rate was reduced by half. The anesthetized mice were now restrained by holding them by the neck. An EDTA-coated hematocrit capillary was inserted at a 30° angle behind the outer left inferior quadrant beyond the right eye bulb and pressed into the blood sinus located behind the upper eyelid with gentle twisting movements. The end of the capillary was passed over a standing 1.5 mL reaction tube containing 5 µL heparin, so that the blood could flow into it. The collected blood was centrifuged at 5,000 × *g* for 5 min. A volume of 150 μL of blood plasma was collected and transferred to a new 1.5 mL reactivity tube. The collected plasma was used directly for further analyses or stored at −80 °C.

### 2.3 Subcutaneous injection in *Mus musculus*

For the application of hormones, a 1 mL syringe was filled with the solution without air bubbles, and the cannula was fixed and vented with the solution. For subcutaneous injection, randomly chosen mice were fixed by grasping the fold of the neck or dorsal skin with the index finger and thumb. The injection cannula was inserted into the skin fold, and a total volume of 100 μL of solution (sesame oil + hormones) was injected per mouse. The administered hormone concentration was 5 μg E2 and 1 mg P4 per animal and was administered daily for 3, 7, or 14 days, depending on the duration of the experiment. The control group received daily injections of sesame oil over the same period.

### 2.4 Ovariectomy

Perioperative analgesia was provided by subcutaneous injection of metamizole (20 mg/kg). Under inhalation anesthesia (2 volume % isoflurane/1 mL O_2_ per minute), the mice were fixed in the prone position. Paramedian to the spine, a 2 cm × 2 cm area was depilated caudally using a disposable razor. Ovariectomy of the mice was performed with the aid of a binocular. Subsequently, a 5 mm incision of the cutis and peritoneum was made 1 cm ventral to the dorsal vertebrae, and, using anatomical forceps, the ovary was pulled out. The ovarian artery and vena ovarica were obliterated by coagulation forceps, and then the ovary and 1 cm of the uterus were removed with spring scissors. The incision of the peritoneum was closed with Prolene suture 6–0 with two stitches. The skin was closed with wound clips (5 mm). Postoperative analgesia was provided with the carprofen (5 mg/kg subcutaneously) every 24 h for 5 days. The success of the procedure was verified by a significant reduction in estrogen and progesterone plasma levels. Experiments were conducted 3 weeks after ovariectomy.

### 2.5 Subcutaneous implantation of hormone pellets in *Mus musculus*

Hormone pellets with biodegradable carrier binder (Innovative Research of America, Sarasota, USA) were used with the following specifications: progesterone (25 mg/pellet, 21-day release), prolactin (7 mg/pellet, 21-day release), and 17β-estradiol (0.1 mg/pellet). Subcutaneous implantation of hormone pellets was performed 3 weeks after ovariectomy. Mice were anesthetized with isofluorane, and a 5 mm cutaneous incision was made cranially at the level of the neck. Anatomic forceps were used to form a skin pocket into which the hormone pellet was implanted. The incision was closed by applying a wound clip, which was removed 14 days after surgery.

### 2.6 Plasma volume determination using Evans blue

Twenty minutes before the Evans blue dye injection, 100 µL heparin was administered interperitoneally to the mice, and 20 µL of 0.4% Evans blue solution was injected into the tail vein. After 1 h, 500 µL of blood was taken from the animals via the ophthalmic sinus, and the plasma was collected by centrifugation and transferred to a new reaction tube. The concentration of Evans blue in the plasma was detected by measuring the OD at 620 nm against a previously prepared standard series. The plasma volume was determined as follows: amount of dye injected (A) / plasma concentration of the dye (C). The hematocrit was determined after puncturing the ocular sinus with a hematocrit capillary using a hematocrit centrifuge. The absolute blood volume was calculated as follows: (plasma volume / hematocrit) × 100 = absolute blood volume.

### 2.7 BrdU incorporation

For BrdU incorporation, pregnant mice received 1 mg/mL BrdU via their drinking water over a period of 7 days. Subsequently, mice were killed, and the hearts were dissected for further analysis. BrdU staining was performed using the BrdU Labeling and Detection Kit II (Roche, Switzerland).

### 2.8 Enzyme-linked immunosorbent assay for quantitative hormone determination

A competitive solid phase enzyme-linked immunosorbent assay (ELISA) was used to determine the concentration of P4 and estradiol in plasma (Estradiol ELISA and P4 ELISA, DRG Instruments GmbH, Germany). For the quantification of P4, serum was used at a dilution of 1:2, sample: standard zero. Plasma hormone concentrations were analyzed photometrically in an Infinite 200 PRO multimode microplate reader, interpolated from a standard curve, and determined using the GraphPad Prism 9.0.1 statistical program.

### 2.9 Electron microscopy analysis

Heparinized adult mice were killed by cervical dislocation, and the hearts were harvested. The hearts were perfused with fixation solution by using a roller pump to administer all solutions at a perfusion flow rate of 4 mL/min. First, the hearts were flushed with a pre-flow solution (0.9% NaCl solution containing 600 μL heparin (5,000 U/mL) per 100 mL solution) for 1 min. This was followed by perfusion with Karnovsky fixative (0.1 M cacodylate buffer containing 4% glutaraldehyde and 6% paraformaldehyde). After fixation, the hearts were removed, the left ventricles isolated, and cut into 2 mm × 2 mm tissue blocks with a scalpel. The blocks were transferred to Karnovsky fixative for secondary fixation and fixed on a shaker at RT by immersion and subsequently stored overnight at 4 °C. Specimens were washed after fixation for 3 × 15 min in cold 0.15 M cacodylate buffer (50 mM cacodylate, 50 mM KCl, 2.5 mM MgCl_2_, pH 7.4) and subsequently fixed for 1 h with 1% osmium tetroxide (buffered with 0.15 M cacodylate buffer) on ice. Afterwards, specimens were washed 15 min with 0.1 M cacodylate buffer and 2 × 15 min with ddH_2_O at RT. Subsequently, specimens were incubated for 60 min in 1% uranyl acetate (in 70% ethanol) and dehydrated in an ascending ethanol series using solutions of 70%, 80%, 90%, 96%, 100%, and 100% ethanol, 15 min each. Specimens were incubated twice in propylene oxide (PO) for 30 min each before incubation in a mixture of PO and Epon 812 (1:1) overnight. The following day, the Epon–PO mixture was substituted with pure Epon 812, and specimens were incubated for 2 h in Epon 812. Specimens were embedded in Epon 812 and kept at 60 °C for 48 h.

For ultrathin sections, 70-nm-thick sections were cut with an ultramicrotome (Ultracut E, Reichert Jung, Germany) and collected on copper or nickel grids. Sections were post-stained with 2.5% uranyl acetate and 0.2% lead citrate and finally analyzed with a LEO AB 912 transmission electron microscope (Carl Zeiss Microscopy GmbH, Germany).

### 2.10 Quantitative real-time PCR

cDNA was generated from 1 µg of total RNA using the SuperScript VILO cDNA Synthesis Kit (Invitrogen, USA) according to the manufacturer's instructions. PCR products were amplified using a RotorGene 6000 (Qiagen, Germany) and the TaqMan Assay-on-Demand system (Applied Biosystems, USA) as described by the manufacturer with the following probes: PGC-1α (Mm 01208835_m1), ERRα (Mm 00433143_m1), VEGF-A (Mm 01281449_m1), and VEGF-B (Mm 00442102_m1). Expression was normalized against 18S rRNA (4318839).

### 2.11 Isolation of adult mouse CMs

Heparinized adult mice were killed by cervical dislocation, and the hearts were dissected. CMs were isolated by Langendorff dissociation. CM contraction was prevented by adding 25 mM 2,3-Butanedione monoxime (BDM) to all used solutions. The enzyme solution was prewarmed to 37 °C by connecting the perfusion system to a water bath. The hearts were cannulated and connected to the perfusion system. After washing with PBS and Ca^2+^-free Tyrode's solution, the hearts were perfused with enzyme solution at a velocity of 2.2 mL/min. The hearts were perfused for 10–13 min, detached from the cannula, atria were disconnected, and ventricles were minced mechanically using forceps. The enzyme reaction was stopped with 5 mL perfusion buffer with 5% FBS, 50 µM CaCl_2_, and the suspension was filtered through a 100 µm cell strainer. Cells were centrifuged for 1 min at 80 × *g*, and the pellet was resuspended in 1 mL Trizol for RNA isolation or fixed for immunocytological studies.

### 2.12 Heart dissection and fixation

Heparinized adult mice were killed by cervical dislocation, and the hearts were dissected. After cannulation of the ascending aorta, the hearts were perfused with PBS, followed by perfusion with 4% PFA and further fixation overnight. The hearts were dehydrated in a 20% sucrose solution and embedded in O.C.T. compound.

### 2.13 Histology, immunofluorescence staining, and microscopy

Using a cryotome CM 3050S (Leica, Germany), 10-µm-thick cryosections were generated from embedded mouse hearts. Staining for collagen was performed by Sirius red staining using standard histology protocols. The following antibodies were used for staining of fixated cells and heart sections (incubation for 2 h at RT in 0.2% Triton X-100 in PBS, 5% donkey serum or overnight at 4 °C in 0.2% Triton X-100 in PBS, 5% donkey serum): ASMAC (1:800, Sigma-Aldrich, Germany), pHH3 Ser 10 (1:100, Chemicon, Germany), Ki-67 (1:400, a gift from J. Gerdes, Forschungszentrum Borstel, Germany), ERRα (1:400, Santa Cruz Biotechnology, USA), PGC1-α (1:50, Santa Cruz Biotechnology, USA), and vimentin (1:800, Chemicon, Germany). Fluorescein *Griffonia simplicifolia* lectin (GSL, Vector Laboratories, USA) and wheat germ agglutinin (WGA, Vector Laboratories, USA) were diluted 1:100 and stained at RT for 1 h. Before Ki-67 staining, antigens were retrieved by boiling the sections in 10 mM sodium citrate buffer, pH 6.0, for 5 min in a microwave at 800 W. After washing, secondary antibodies conjugated to Cy2, Cy3, and Cy5 (1:400, Jackson ImmunoResearch, USA), diluted in 1 μg/mL Hoechst 33,342 (nuclear stain) in PBS, were applied for 1 h at room temperature. Sections were mounted with coverslips and imaged using an inverted fluorescence microscope (Axiovert 200M, Carl Zeiss, Germany) with filters for DAPI, GFP, Cy3, and Cy5; 25×, 40×, and differential interference contrast (DIC) plan apochromat oil objectives; an ebx 75 light source; and an AxioCam MRm digital camera and AxioVision Rel. 4.8 Software (Zeiss, Germany).

### 2.14 Determination of capillary density and hypertrophy

In this work, the lectins GSL-I and WGA coupled with a fluorescent dye were used to determine the capillary density in skeletal and cardiac muscle as well as to analyze the cross-sectional area (CSA) of cardiomyocytes (hypertrophy). The vessels were stained by GSL-I and cell membranes by WGA as mentioned above. In a humid incubation chamber, the sections were outlined with a grease pencil and rehydrated with PBS for 5 min. The lectins were diluted in 1 μg/mL Hoechst 33342 in PBS (1:100), applied to the tissue sections, and incubated for 1 h at RT. In the absence of light, the tissue sections were washed three times with PBS for 5 min each time. The sections were then washed with ddH_2_O for 5 min, covered with Fluka mounting medium and coverslips, and dried for 12 h at RT. The tissue sections were stored at 4 °C until analysis on an Axiovert 200M fluorescence microscope. The images were analyzed without knowledge of the test group assignment and thus blinded. The cross-sectional area of the cardiomyocytes was quantified using the contour function of the image analysis program AxioVision Rel 4.8. The number of cardiomyocytes and capillaries per field of view (40× objective) was determined using ImageJ 1.39u software, and the capillary density per cardiomyocyte was calculated.

### 2.15 Statistics

All statistical analyses were performed and presented using the statistical program GraphPad Prism 9.0.1. Data were expressed as mean ± SEM and were analyzed with an unpaired two-tailed *t*-test or one-way analysis of variance (ANOVA) with Dunnett's multiple comparisons. *P*-values of ≤0.05, ≤0.01, ≤0.001, and ≤0.0001 were considered statistically significant, very significant, and highly significant, respectively. Equality of group variances was tested for by the Brown–Forsythe test.

All measurements were taken from distinct samples. The minimal sample size for all mouse studies was determined by a power analysis using the PS Power and Sample Size Calculations program V3.1.6 from Vanderbilt University (http://biostat.mc.vanderbilt.edu/PowerSampleSize) with the following assumptions: We are planning a study of a continuous response variable from independent control and experimental subjects with one control per experimental subject. In a previous study, the response within each subject group was normally distributed with a standard deviation of 0.1. If the true difference in the experimental and control means is 0.3, we will need to study three experimental subjects and three control subjects to be able to reject the null hypothesis that the population means of the experimental and control groups are equal with probability (power) 0.8. The Type I error probability associated with this test of the null hypothesis is 0.05.

### 2.16 Study approval

All applicable international, national, and/or institutional guidelines for the care and use of animals were followed. All procedures performed in studies involving animals were in accordance with the ethical standards of the institution at which the studies were conducted and were approved by the responsible governmental animal care and use office (NTP 84-02.04.2015.A118).

## 3 Results

### 3.1 Reversible cardiac hypertrophy and increased capillary density during pregnancy

**Figure 1:**
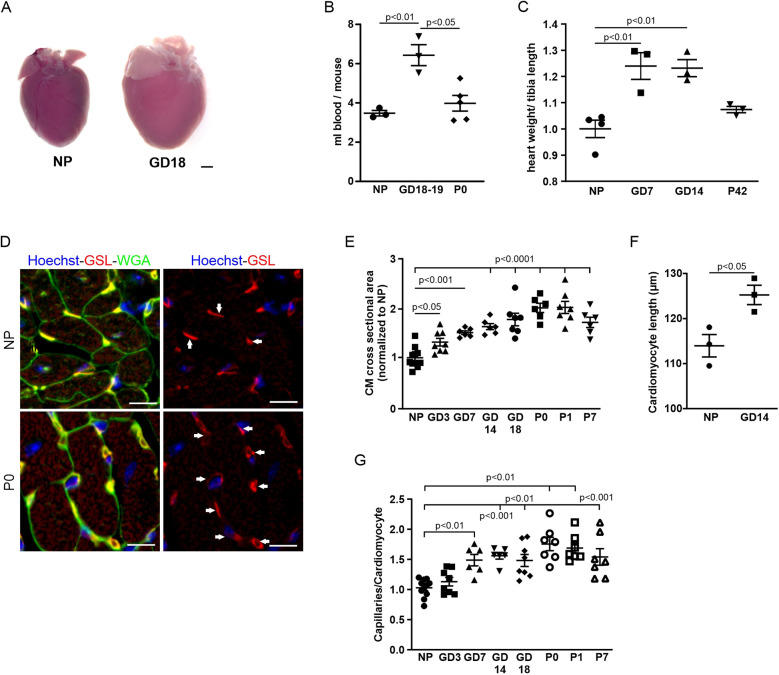
Cardiovascular adaptations during pregnancy. **(A)** Representative hearts from an NP and a GD18 mouse. Scale bar = 1 mm. **(B)** Blood volume was twofold higher in late pregnancy compared with NPs and after delivery (P0). *n* = 3−5 mice per group. **(C)** Quantitation of hypertrophy, as measured by heart weight/tibia length. *n* = 3–4 mice per group. Statistical significance was assessed using a one-way ANOVA with Dunnett's test for multiple comparison. **(D)** Immunostaining of cardiac sections from NP and P0 mice with GSL-I, indicating capillaries (arrows), and WGA for cell borders; nuclei are stained with Hoechst (blue). Scale bar = 10 µm. **(E)** Quantitation of hypertrophy as cross-sectional area (CSA). *n* = 6–10 mice per group. Statistical significance was assessed using a one-way ANOVA with Dunnett's test for multiple comparison. **(F)** Quantitation of CM length of isolated cells from NP and GD 14 mice (*n* = 3 mice per group, *n* = 30 cells). **(G)** Quantitation of capillary density as capillaries per cardiomyocyte. *n* = 6–10 mice per group. Data are presented as mean ± SEM. Statistical significance was assessed using a one-way ANOVA with Dunnett's test for multiple comparison.

We focused our analysis on two aspects of cardiovascular adaptation during pregnancy: increased heart weight due to cardiac hypertrophy and capillarization. The hearts of CD-1 mice were analyzed throughout pregnancy and 1 week postnatally. As expected, overall heart size was remarkably increased in late pregnancy (GD18) compared with the hearts from NP mice (Figure 1A). As cardiac hypertrophy during pregnancy is commonly attributed to an increase in blood volume causing increased preload, we determined the blood volume in NP mice in late pregnancy and postpartum. We found that the plasma volume was significantly increased in late pregnancy and returned to non-pregnant levels postpartum (Figure 1B). Cardiac dry weight was significantly increased as early as gestational day 7 (GD7) and stayed elevated compared with NP control hearts until delivery (Figure 1C). Six weeks after giving birth, cardiac weight dropped back to that of the control group. Hypertrophy of CMs was visible in cardiac cross sections stained with WGA to mark cell membranes (Figure 1D). At the cellular level, hypertrophy was assessed by measuring CM cross-sectional area (CSA) and length, and it was revealed that CSA increased during pregnancy, becoming significant at GD7 and starting to revert at P7 (Figures 1E,F). CM length was significantly increased at GD14 (Figure 1F).

As adaptive hypertrophy leads to increased metabolic demand and thus blood perfusion, we also assessed capillary density in the heart by quantifying the ratio of GSL-stained capillaries per CM in heart sections of pregnant and NP mice (Figure 1D). A significant increase in capillary density was observed starting at GD7 and reached a peak at P0 (Figure 1G).

### 3.2 Cardiac cell proliferation during pregnancy

**Figure 2:**
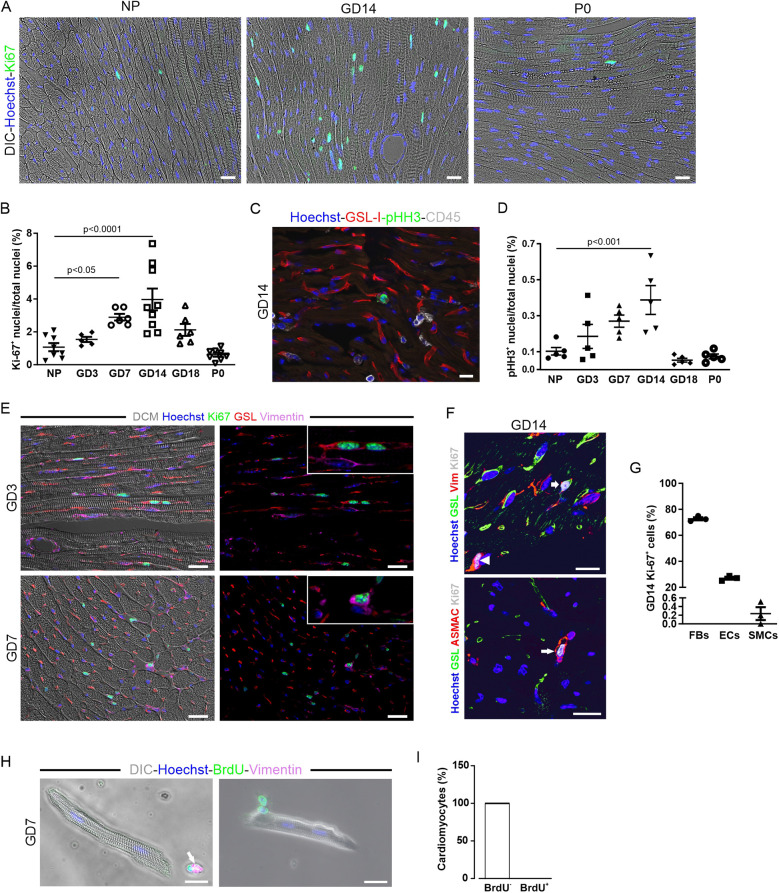
Increased proliferation of cardiac cells over the course of pregnancy. **(A–D)** Proliferation as determined by Ki-67 **(A)** or pHH3 immunostaining **(C)**; nuclei are stained with Hoechst (blue). Scale bar = 50 µm. **(B,D)** Quantitation of Ki-67^+^ and pHH3^+^ nuclei during pregnancy, postnatally, and in NP controls. NP, non-pregnant; GD, gestational day; P, postnatal day. *n* = 5–9 mice per group. Statistical significance was assessed using a one-way ANOVA with Dunnett's test for multiple comparison. **(E)** Immunofluorescence analysis of cardiac sections from mice at early (GD3, upper picture) and midterm pregnancy (GD7, lower picture). Scale bar = 20 µm. **(F)** Representative fluorescence images showing Ki-67^+^ EC (GSL, vimentin, arrow), fibroblast (vimentin, arrowhead), and smooth muscle cell (ASMAC, long arrow) at GD14; nuclei are stained with Hoechst (blue). Scale bar = 20 µm. **(G)** Quantitation of Ki-67^+^ ECs (GSL^+^), fibroblasts (FBs, vimentin^+^), and smooth muscle cells (SMCs, a-smac^+^) at GD14, *n* = 3. **(H,I)** Pregnant mice received 1 mg/mL BrdU via the drinking water for a period of 1 week. **(H)** isolated and fixed CMs analyzed for BrdU integration by immunofluorescence staining; 100% of the CMs analyzed were found to be BrdU^−^ (*n* = 2,253). **(I)** In co-stainings, BrdU^+^ cells additionally expressed vimentin (H, arrow) and are therefore non-cardiomyocytes. DIC, differential interference contrast. Data are represented as mean ± SEM, scale bar = 20 µm.

Given that pregnancy promotes neurogenesis in the brain (23), we wondered whether it also induces cardiac cell proliferation. To investigate this, we stained heart sections from pregnant mice with the mitotic marker Ki-67 (Figure 2A). Cardiac sections from GD14 mice stained with Ki-67 showed multiple Ki-67-positive nuclei, while in NP controls, only a few nuclei were positive (Figure 2A). Surprisingly, this stopped shortly after P0, after the mice had given birth (Figure 2A). Quantification of Ki-67-positive cardiac nuclei revealed an increase during pregnancy, peaking at GD14, and a decrease thereafter (Figure 2B). This was confirmed using the specific M-phase marker pHH3 (Figures 2C,D).

Our data suggest an influence of pregnancy hormones such as E2 and P4, whose levels rise during pregnancy (2), on the mitotic activity of cardiac cells. As hormone fluctuations also occur during the female estrus cycle, we tested this aspect in more detail. We found a constant number of Ki-67^+^ cardiac cells during the proestrus and estrus phases, but a maximum number during the diestrus phase, when P4 levels peaked (Supplementary Figure S1A). These data strongly suggested an influence of E2 and P4, the most dynamically fluctuating steroid hormones during estrus, on the cell cycle activity of cardiac cells.

### 3.3 ECs, fibroblasts, and smooth muscle cells proliferate, but not CMs

To identify the proliferating cell types in the heart, we co-stained with different markers: GSL-I and vimentin for ECs, cross striations in brightfield for CMs, α-smooth muscle actin (α-SMA) and vimentin for smooth muscle cells, and vimentin alone for fibroblasts at different gestational days (Figures 2E,F). Quantification at GD14 revealed that most Ki-67-positive cells were fibroblasts (72.7 ± 1.1%) and ECs (27.0 ± 1.1%) (Figure 2G). Smooth muscle cells accounted for only a small fraction (0.2 ± 0.1%) of the total cells in the cell cycle, and no Ki-67-positive CMs were detected. Some Ki-67 positive nuclei, which were unequivocally identified as non-CMs by cell type-specific markers, colocalized with CM cross striation visualized by either (DIC) or α-actinin staining (Figure 2E), highlighting the challenges in identifying CM nuclei in cardiac tissue sections. To detect a potentially small, slowly cycling pool of CMs that could be missed by histological analysis, pregnant mice were given BrdU in their drinking water during the first week of pregnancy. Single CMs were isolated from the hearts of those mice by Langendorff dissociation and stained for the presence of BrdU (Figure 2H). DNA replication in CMs, indicated by the incorporation of this thymidine analog, was not detectable (Figure 2I), proving that cell cycle activity in CMs was not increased during pregnancy.

### 3.4 Capillary density increases after treatment with P4 and E2 in the absence of cardiac hypertrophy

**Figure 3:**
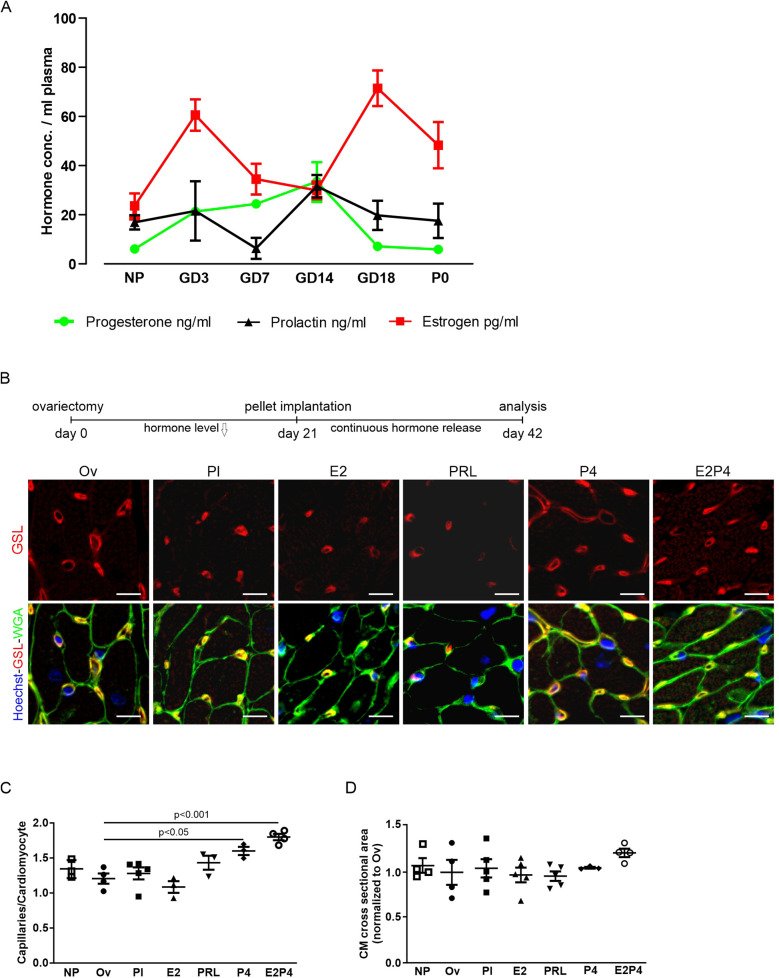
Hormone treatment increases capillary density in hearts. **(A)** Plasma levels of P4, prolactin, and E2 at different gestational and postnatal days as determined by ELISA. *n* = 5–9 mice per group. **(B)** Analysis of capillary density using immunofluorescence staining for GSL-I and WGA of adult heart sections after hormone treatment by pellet implantation in ovariectomized mice. Scale bar = 10 µm. **(C,D)** Quantification of capillary density and hypertrophy from **(B)**, as the ratio of capillaries per CM **(C)**, or normalized CM cross-sectional area **(D)**. *n* = 3–5 mice per group. Statistical significance was assessed using a one-way ANOVA with Dunnett's test for multiple comparison. Ov, ovariectomized; Pl, placebo; PRL, prolactin; P4, progesterone; E2, estrogen; E2P4, estrogen + progesterone.

To identify the pregnancy hormones underlying cardiac adaptation and to mimic their effects *in vivo*, we measured hormone levels in pregnant CD-1 mice and correlated them with cell cycle activity. Levels of E2 rose strongly in early pregnancy, declined in mid-pregnancy, peaked at GD18, and returned to NP levels in late pregnancy and postpartum (Figure 3A). The plasma levels of P4 and prolactin increased up to GD14 and decreased thereafter. Comparison with the Ki-67 profile revealed that the blood levels of P4 and prolactin correlated most closely with the changes in the proliferation rate of the cardiac cells. Next, we explored whether applying pregnancy hormones, which mimic the hormonal profile during pregnancy, could induce the observed increase in capillary density.

To this end, hormone-releasing pellets were implanted into ovariectomized mice 2 weeks after surgery. As a direct readout of EC proliferation, capillary density was determined after 3 weeks of pellet treatment by staining with WGA and GSL-I (Figure 3B). While pellets, releasing either E2 or prolactin, had no effect on capillary density compared with placebo, pellets releasing P4 alone or a combination of E2 and P4 led to a significant increase in heart capillarization from 1.28 ± 0.09 (placebo) to 1.60 ± 0.06 (P4) and 1.80 ± 0.05 (E2 + P4) capillaries per CM (Figure 3C). The effect was more pronounced in mice receiving both E2 and P4 for 3 weeks compared with P4 alone. Interestingly, no increase in CM surface area in cross sections could be observed in either condition, indicating that pregnancy-induced capillary proliferation is not sufficient to also drive cardiac hypertrophy (Figure 3D). Thus, increases in capillary density and cardiac hypertrophy appear to be two independent adaptive mechanisms.

### 3.5 Optimized hormone treatment protocol increases capillary density in female and male mice independent of hypertrophy

From the hormone treatment experiment using implanted pellets, it was evident that the most substantial increase in capillary density was observed after the application of a combination of E2 and P4. However, determination of the plasma hormone levels in those mice revealed a steady decrease in P4 and high variability, despite the theoretical continuous release from the implanted pellets (Supplementary Figure S1D). This was due to occasional encapsulation of the pellets in fibrous tissue, which prevented them from releasing the hormones to their full potential. To achieve more consistent hormone plasma levels, we injected female mice daily with 5 µg E2, 1 mg P4, or a combination of both for 14 days. Capillary density, determined by staining for WGA and GSL-I (Supplementary Figure S2A), revealed that the combined treatment with E2 and P4 had the strongest effect (Supplementary Figure S2B) and also caused the highest plasma levels of E2 and P4 (Supplementary Figures S2C,D), reaching 62% of the GD14 physiological levels for P4% and 101% for E2 during pregnancy.

**Figure 4:**
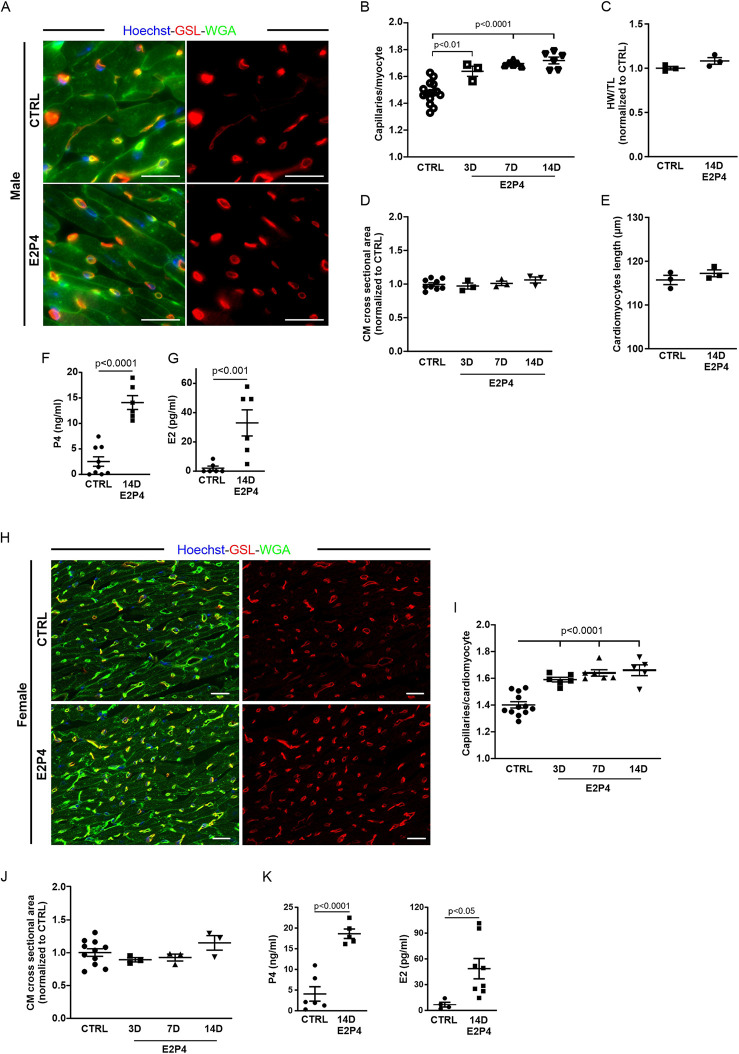
E2P4 treatment increases the capillary density in the hearts of adult female and male mice. **(A)** Mice were injected daily subcutaneously with a mixed solution of P4 and E2 for 3, 7, or 14 days. Capillary density was determined as the ratio of capillaries per CM by immunostaining of cardiac sections for GSL-I (red) and the CM membrane with WGA (green); nuclei are stained with Hoechst (blue). Scale bar = 20 µm. **(B)** Quantitation of capillary density in male mice after 3, 7, and 14 days of E2P4 treatment. *n* = 3–12 mice per group. **(C)** Quantitation of hypertrophy, as measured by heart weight/tibia length. *n* = 3 mice per group. Statistical significance was assessed using a one-way ANOVA with Dunnett's test for multiple comparison. **(D)** Quantitation of CM hypertrophy as assessed by the cross-sectional area of CMs. *n* = 3–12 mice per group. Statistical significance was assessed using a one-way ANOVA with Dunnett's test for multiple comparison. E2P4: estrogen + progesterone. **(E)** Quantitation of CM length of isolated cells from NP and GD 14 mice (*n* = 3 mice per group, *n* = 30 cells). **(F,G)** ELISA analysis of P4 **(F)** and E2 **(G)** concentrations in blood plasma (ng/mL plasma) after 14 days of treatment (sesame oil (control) and E2P4). *n* = 6–9 mice per group. Unpaired *t*-test (two-sided). **(H)** Capillary density in E2P4-treated female mice was determined as the ratio of capillaries per CM by immunostaining of cardiac sections for GSL-I (red) and the CM membrane with WGA (green); nuclei are stained with Hoechst (blue). Scale bar = 20 µm. **(I)** Quantitation of capillary density in male mice after 3, 7, and 14 days of E2P4 treatment. **(J)** Quantitation of CM hypertrophy as assessed by the normalized cross-sectional area of CMs. Statistical significance was assessed using a one-way ANOVA with Dunnett's test for multiple comparison. E2P4: estrogen + progesterone. *n* = 3–12. **(K)** ELISA analysis of P4 and E2 concentrations in blood plasma (ng/mL plasma) after 14 days of treatment [sesame oil (control) and E2P4]. *n* = 4–8. Unpaired *t*-test (two-sided). Data correspond to mean ± SEM.

According to this result, we changed our treatment protocol to daily injections of 5 µg E2 and 1 mg P4 (E2P4) over 14 days. To test whether this hormone regimen could also induce capillary proliferation in male mice, they were treated with daily injections for 14 days (Figure 4). Staining of cardiac sections for WGA and GSL-I (Figure 4A) revealed a significant increase in capillary density in male mice (Figure 4B). As in the experiments with the pellet implantation, there was no sign of cardiac hypertrophy, since the heart weight to tibia length ratio, the cross-sectional area of CMs, and the CM length were not increased compared with the controls (Figures 4C–E). P4 plasma levels were higher than 10 ng/mL after treatment in male mice (Figure 4F), representing a fivefold increase compared with controls. Likewise, E2 plasma levels were significantly increased (Figure 4G). This result indicated that the effect was sex-independent, as it was evident in female and male mice after 14 days of treatment (Figures 4H–K). To understand the dynamics of this increase in capillary density, female and male mice were treated for either 3 or 7 days with E2P4. As early as 3 days after treatment, a significant increase was evident in treated male mice (Figure 4B) and, after 7 days, in female mice (Figure 4I). Again, this rise in capillary density was not accompanied by cardiac hypertrophy, as measurements of the cross-sectional area of CMs did not differ significantly from those of controls (Figures 4D,J).

**Figure 5:**
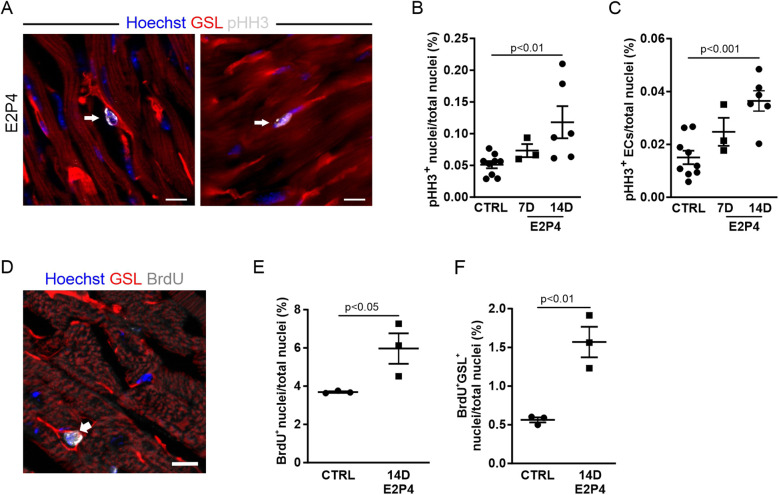
EC proliferation is increased after 14 days of E2P4 hormone treatment. **(A)** Co-staining for GSL-I (red) and pHH3 (white) for detection of the proliferation of ECs (arrow) and other cell types (arrowhead) in the heart; nuclei are stained with Hoechst (blue). Scale bar = 10 µm. Quantitation of proliferating ECs **(B)** and other cardiac cell types **(C)** in relation to the total number of nuclei in heart sections from control and 7D and 14D mice (*n* = 3–9 mice per group). Statistical significance was assessed using a one-way ANOVA with Dunnett's test for multiple comparison. **(D)** The co-staining for GSL (red) and BrdU (white) marks an EC after or in the S phase (white arrow) in the heart; nuclei are stained with Hoechst (blue). Scale bar = 10 µm. **(E,F)** Quantitation of BrdU-positive cell nuclei of ECs **(E)** and cardiac cells **(F)** in the heart sections of hormone-treated mice. *n* = 3 mice for each group. Unpaired *t*-test (two-sided). Data correspond to mean ± SEM.

The observed increase in capillary density could be due to EC proliferation, and we therefore analyzed the proliferation rate by immunostaining for pHH3 (Figure 5A). After 7 days of treatment, an increase in pHH3^+^ cardiac cells (Figure 5B) and ECs (Figure 5C) in the hearts of treated mice was observed. After 14 days of treatment, the increase in total proliferative cardiac cells (Figure 5B) and, particularly in ECs (Figure 5C), became statistically significant, recapitulating our observation in the hearts of pregnant mice.

To further verify this finding, osmotic minipumps filled with BrdU solution were implanted subcutaneously in each animal before the start of the experiment. The incorporation of BrdU during the S phase of the cell cycle allowed for the study of DNA synthesis activity over the course of the 14-day treatment. Cardiac sections were labeled with antibody staining against BrdU and GSL-I for visualization of ECs (Figure 5D). Quantification of BrdU-positive nuclei revealed a significant increase in cardiac cells with DNA synthesis activity after hormone treatment (Figure 5E). The proportion of BrdU-positive ECs (identified by GSL staining) increased by 2.8-fold (Figure 5F). Overall, approximately 26% of all BrdU-positive cells were ECs, whereas most BrdU-positive cells were probably fibroblasts, as seen in the hearts of GD14 mice (Figure 2G). The observed rise in EC proliferation during hormone treatment correlated with the increased capillary density in the myocardium, indicating that E2P4 treatment directly or indirectly enhanced EC proliferation in the heart.

To test whether our hormone treatment was specific to the heart or affected other striated muscles, we dissected the tibialis anterior muscle and diaphragm of male mice treated with E2P4 for 14 days and determined capillary density, as was done for heart sections (Supplementary Figures S3A,B). Interestingly, we could not detect an increase in capillary density in any of the analyzed skeletal muscles (Supplementary Figure S3C), suggesting that the observed effect after E2P4 treatment is restricted to the heart.

### 3.6 The increased capillary density by hormone treatment is not mediated by signaling through nuclear P4 or E2 receptors

**Figure 6:**
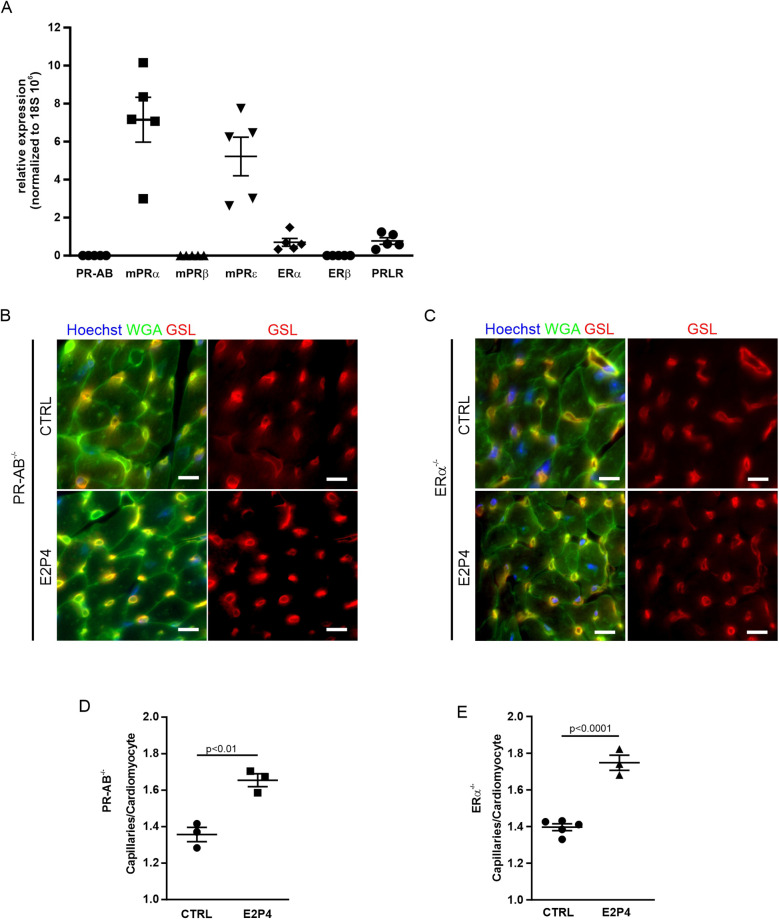
Increase of capillary density in PR-AB^−/−^ and ERα^−/−^ mice after E2P4 treatment. **(A)** Overview of qPCR results with specific probes for progesterone receptors, estrogen receptors, and prolactin receptors in the hearts of non-pregnant mice. Shown are the relative expression differences, normalized to 18S RNA. *n* = 5 mice per group. **(B,C)** Immunofluorescence staining of tissue sections of the hearts from non-pregnant female PR-AB^−/−^ **(B)** or non-pregnant female ERα^−/−^ mice **(C)** after 14 days of sesame oil (CTRL) or E2P4 treatment (E2P4) for GSL (red) and WGA (green); nuclei are stained with Hoechst (blue). Scale bar = 10 µm. **(D,E)** Quantitation of capillaries per CM of E2P4-treated PR-AB^−/−^ **(D)**, *n* = 3 mice per group, or ERα^−/−^ mice **(E)**, *n* = 3–5 mice per group. Unpaired *t*-test (two-sided). Data correspond to mean ± SEM.

As a combination of E2 and P4 induces EC proliferation, resulting in increased capillary density in the heart, we sought to unravel the underlying signaling mechanism. Both hormones act mainly through nuclear receptors. To determine which hormone receptors are expressed in the female mouse heart, we performed quantitative real-time PCR (qPCR). Of the nuclear receptors, neither the P4 receptor isoforms PR-A and PR-B nor ERβ expression was detectable, whereas small amounts of ERα were evident (Figure 6A). Among the non-nuclear receptors, the membrane P4 receptors mPRα and mPRε were expressed as well as the prolactin receptor (PRLR) (Figure 6A).

To explore the impact of the nuclear receptors PR-A, PR-B, and ERα, we treated PR- and ERα-deficient mice with our hormone regimen for 14 days and determined cardiac capillary density (Figures 6B,C). We found that capillary density was increased in the hearts of PR- as well as ERα-deficient mice after E2P4 treatment (Figures 6B,C), ruling out the involvement of either of these two nuclear receptors. In addition, to investigate the involvement of PR signaling in the induction of EC proliferation, we used the PR-lacZ mouse strain to detect PR expression in the heart. There was no PR expression in mouse hearts, but, as expected, PR expression was detected in the uterus, which was used as a positive control (Supplementary Figure S3D), thereby confirming the results from the qPCR analysis.

### 3.7 Upregulation of angiogenic factors and their receptors during pregnancy and after hormone treatment

**Figure 7:**
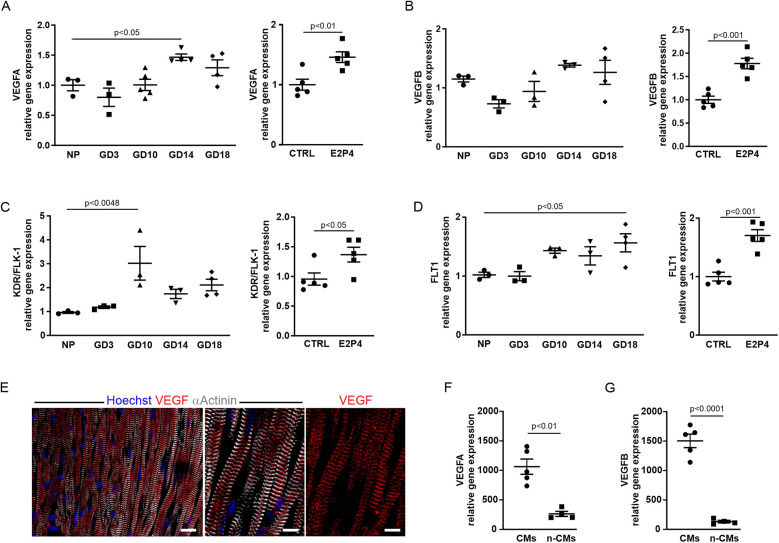
The expression of angiogenic factors increases after E2P4 treatment. **(A–D)** Overview of qPCR results with probes for VEGF-A **(A)** and VEGF-B **(B)** and their receptors KDR/FLK-1 **(C)**, and FLT1 **(D)** for the hearts from non-pregnant controls, GD3, GD10, GD14, and GD18 mice, or after 14 days of treatment with E2P4. Shown are relative expression differences normalized to 18S RNA and relative to non-pregnant or sesame oil-treated controls (CTRL). *n* = 3–5 mice per factor and receptor. Statistical significance was assessed using a one-way ANOVA with Dunnett's test for multiple comparison or unpaired *t*-test (two-sided). **(E)** Immunofluorescence staining for α-actinin (white) and VEGF (red) indicates localization of VEGF to the Z-discs of CMs. Scale bar = 20 μm. Detailed images: Scale bar = 10 μm. **(F,G)** Quantitation of relative expression levels of VEGF-A **(F)** and VEGF-B **(G)** in isolated CMs (CMs) and other cardiac cell types (n-CMs). *n* = 4–5 mice per group, unpaired *t*-test (two-sided). Data correspond to mean ± SEM.

To gain insight into the mechanisms that lead to increased cardiac cell proliferation, we performed expression analysis of known pro-angiogenic genes by qPCR in the hearts from pregnant and non-pregnant mice treated for 14 days with E2P4. This revealed an increase in VEGF-A (Figure 7A) and VEGF-B (Figure 7B) and their receptors KDR/Flk-1 (Figure 7C) and FLT-1 (Figure 7D) over the course of pregnancy as well as after E2P4 hormone treatment. In pregnant mice, VEGF-A and FLT1 showed a statistically significant increase (Figures 7A,D), especially in the last third of pregnancy, while KDR/Flk-1 was increased at mid-pregnancy (Figure 7C). VEGF-B showed a tendency to increase, but did not reach statistical significance (Figure 7B). However, hormone treatment led to a significant increase in all four factors after 14 days (Figures 7A–D). VEGFs are potent angiogenic factors, and therefore, we wanted to determine the cellular source of VEGF production in mouse hearts. Immunofluorescence staining of cardiac sections from 10-week-old CD-1 mice showed expression of VEGF-A in α-actinin^+^ CMs (Figure 7E). To identify the cellular source of VEGF-A and VEGF-B, the hearts from CD-1 mice were either dissociated with CM depletion or CMs isolated by Langendorff dissociation. RNA was prepared from both CMs and non-CMs, and qPCR analysis was performed. Although both CMs and non-CMs expressed VEGF-A and B, CMs were the major source of these angiogenic factors (Figures 7F,G).

VEGF-A has been reported to induce increased capillary permeability (24), and we therefore examined transmission electron micrographs of heart sections from treated male mice (14 days E2P4) and a pregnant mouse (GD14). The capillaries in the heart sections of the control mice exhibited the typical structure of the capillary wall (Supplementary Figure S4A, top) with a continuous endothelium (green arrow) and adjacent pericytes (white arrows) surrounded by the basal lamina (red arrows). In contrast, significant structural changes in the capillaries were observed in the heart sections of the pregnant mouse (Supplementary Figure S4A, bottom left). The endothelium of these capillaries exhibited marked fenestration (black arrows), indicative of increased capillary permeability. In addition, the pericytes (white arrow) were not fully enclosed by the basal lamina (red arrows). In the hearts of E2P4-treated animals, both structurally intact capillaries (Supplementary Figure S4B, top) and capillaries exhibiting distinct morphological alterations were detected (Supplementary Figure S4B, bottom). These capillaries displayed marked endothelial fenestration. Together with the pericytes (white arrow), they were only partially enclosed by a basal lamina, which was focally discontinuous or entirely absent (red arrow), closely resembling the capillaries in the hearts of pregnant mice. Accordingly, capillaries in the hearts of both E2P4-treated and pregnant mice exhibited morphological features consistent with VEGF-A-driven angiogenesis.

### 3.8 Hormone treatment increases capillary density through the peroxisome proliferator-activated receptor gamma coactivator 1-alpha (PGC-1α) pathway

**Figure 8:**
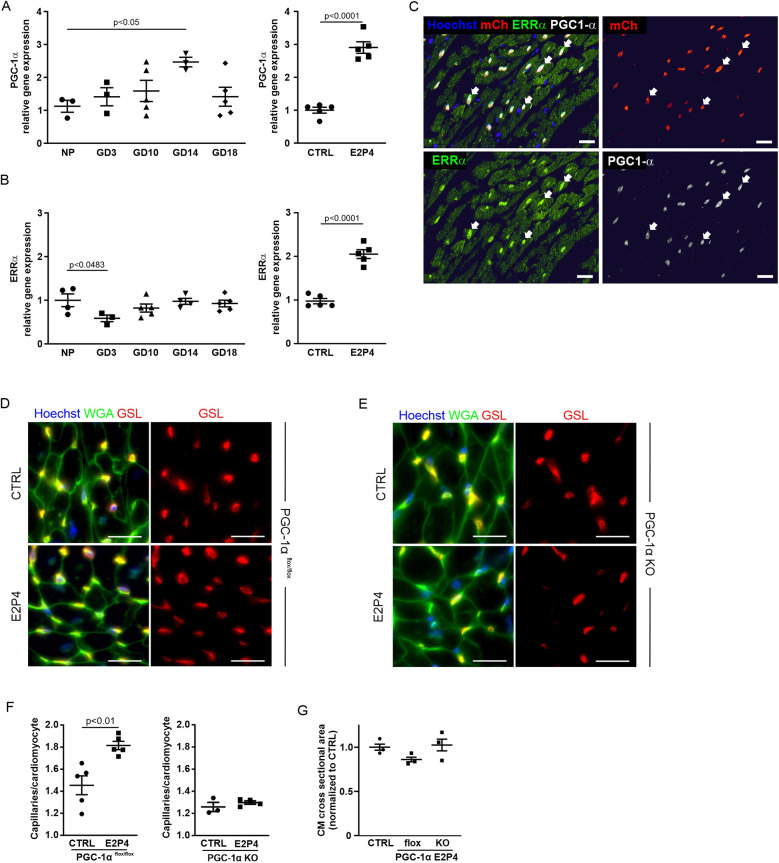
Hormone treatment increases capillary density through the PGC-1α pathway. **(A–B)** Overview of qPCR results with probes for PGC-1α **(A)** and ERRα **(B)** for the hearts from non-pregnant controls, GD3, GD10, GD14, and GD18 mice, or after 14 days of treatment with E2P4. Shown are relative expression differences normalized to 18S RNA and relative to non-pregnant or sesame oil-treated controls. *n* = 3–5 mice per factor and receptor. Statistical significance was assessed using a one-way ANOVA with Dunnett's test for multiple comparison or unpaired *t*-test (two-sided). **(C)** Immunofluorescence stainings for PGC-1α (white) and ERRα (green) in heart sections of αMHC-H2B-mCherry mice indicate colocalization (arrows) of the proteins in CM cell nuclei (red). Cell nuclei are stained with Hoechst (blue). Scale bar = 20 μm. **(D,E)** Immunofluorescence staining with GSL (red) and WGA (green) of tissue sections of the hearts from PGC-1α^−/−^ **(D)** and **(E)** PGC-1α^flox/flox^ mice after a 14-day sesame oil (CTRL) or E2P4 treatment (E2P4). Cell nuclei are stained with Hoechst (blue). Scale bar = 20 µm. **(F)** Quantitation of capillaries per CM in PGC-1α^flox/flox^ and PGC-1α KO mice; *n* = 3–5 mice per group. **(G)** Quantitation of CM hypertrophy as assessed by the normalized cross-sectional area of CMs; *n* = 3 mice per group. Data correspond to mean ± SEM.

VEGF plays a key role in angiogenesis and capillarization (24). Since PGC-1α/ERRα signaling has been shown to induce VEGF expression in CMs, the primary cell source for VEGF production in the heart (21), we next examined the involvement of this pathway. PGC-1α is a transcriptional coactivator that can interact with the orphan nuclear receptor ERRα to induce transcription of its target genes such as VEGF-A (21). qPCR analysis revealed an increase in PGC-1α over the course of pregnancy, peaking at GD14 and after 14 days of E2P4 hormone treatment (Figure 8A).

ERRα expression was downregulated during early pregnancy and remained at a control level over the course of pregnancy (Figure 8B). However, ERRα was significantly upregulated after 14 days of E2P4 treatment (Figure 8B). Immunostaining for ERRα of cardiac sections from H2B-mCherry mice, a strain in which all CM nuclei are marked by mCherry fluorescence (25), demonstrated nuclear localization of ERRα in CM. PGC1-α was co-expressed in CM nuclei together with ERRα (Figure 8C). These data indicated a possible connection between the PGC1α/ERRα pathway and VEGF production in CMs, which could be the cause for the increased capillary density after E2P4 treatment.

To test the involvement of the PGC-1α/ERRα pathway in cardiac capillarization, we employed a CM-specific PGC-1α knockout mouse model (21). In this, a floxed PGC-1α gene is CM-specifically knocked out by an αMHC-Cre expression construct. We treated male αMHC-Cre/PGC-1α^flox/flox^ and PGC-1α^flox/flox^ mice with daily injections of E2P4 for 2 weeks and determined capillary density and CM size (Figures 8D–G). While a significant increase in capillary density was noted for PGC-1α^flox/flox^ controls (Figures 8D,F), there was no change in the capillary density in the hearts from PGC-1α knockout mice after hormone treatment (Figures 8E,F). These data demonstrate that activation of the PGC-1α/ERRα pathway in CMs is required for hormone-induced capillary expansion, likely via the secretion of VEGF by CMs. In general, compared with PGC-1α^flox/flox^ controls, PGC-1α knockout mice exhibited decreased capillarization of the heart. This effect has already been described before (21), together with a tendency for hypertrophy, which manifests after the first pregnancy. We confirmed an overall increase in CM area in the hearts of PGC-1α knockout mice (Figure 8G). Together, these findings establish a CM-intrinsic, PGC-1α/ERRα-dependent mechanism by which E2P4 treatment enhances myocardial capillarization, independently of hypertrophic remodeling.

## 4 Discussion

During pregnancy, the maternal heart responds to increased cardiac demands with increased capillarization and reversible hypertrophy. We wondered by which mechanisms these adaptations are regulated and focused on cardiac proliferation, hypertrophy, and hormonal changes during pregnancy. The percentage of proliferating cells and capillary density in the heart increased until GD3 compared with the control group, peaked at GD14, and ceased immediately after delivery. This was accompanied by an increase in CM hypertrophy. Given the elevation of E2 and P4 levels during pregnancy, we focused on these pregnancy hormones and their signaling pathways that underlie the pregnancy-induced increase in cardiac capillarization.

To mimic the action of pregnancy hormones, we show that treatment with P4 and E2 affects EC proliferation and vascularization in the hearts of adult female and male mice, but not CM size. Interestingly, this effect was cardiac-specific, as skeletal muscles such as the diaphragm or tibialis anterior did not show an increase in capillarization after the hormone treatment. Hormone-dependent regulation of proliferation and differentiation is known for various cell types during pregnancy. In the subventricular zone of the forebrain, for example, the formation of neuronal progenitor cells during pregnancy has been demonstrated (26). P4 has been shown to stimulate EC proliferation in the endometrium during pregnancy and endometrial angiogenesis after P4 injections in ovariectomized mice (27). In addition, transcriptome and proteome analyses of the hearts of pregnant mice recently revealed an increase in genes associated with cell proliferation, cytokinesis, and angiogenesis (28), consistent with our findings. In our setting, we demonstrated an increase in the proliferation of cardiac ECs during pregnancy and after P4 treatment using three different methods (Ki-67, pHH3, BrdU), all of which gave similar results. The effect of P4 was maximized when combined with E2, and treatment caused a significant increase in proliferation as early as 3 days after hormone application and further increased until day 14, which was the endpoint of our study. This time point was chosen because endothelial proliferation peaked during pregnancy at GD14 and declined postpartum. The fast increase in proliferative ECs after E2P4 treatment and the rapid regression of this effect on day P0 prove that this is a rapid response. This is characteristic of steroid hormones such as E2 and P4 (29, 30), and the rapid kinetics indicate that hypervolemia was not the underlying cause of cardiac hypertrophy and EC proliferation. In fact, co-application of E2 and P4 did not induce cardiac hypertrophy.

Next, we investigated the signaling pathway associated with the increase in capillary density triggered by pregnancy hormones. Since the expression of VEGF and its receptors increased in the hearts of pregnant mice and after steroid treatment, we focused on its regulation. VEGF is a crucial stimulator of new vessel formation, essential for forming new capillaries through angiogenic sprouting (31). Loss of cardiac VEGF leads to reduced myocardial capillarization and subsequently to ischemic cardiomyopathy (32). It has been shown that the increase in cardiac angiogenesis during pregnancy is associated with increased gene expression of VEGF (9, 10). We detected an E2P4-induced expression of VEGF and its receptors, suggesting a similar VEGF-driven mechanism. This was corroborated by the morphology of capillaries in the hearts of pregnant and hormone-treated mice, which exhibited features characteristic of increased vascular permeability as mediated by VEGF (33). As the hormone-induced expression of VEGF correlated with increased expression of PGC-1α and ERRα, we focused on this pathway as the most likely regulator of VEGF signaling. PGC-1α is a potential mediator of angiogenesis that controls VEGF expression and secretion in skeletal muscle by coactivating ERRα (22). It has been shown in CM-specific PGC-1α knockout mice that loss of this program results in decreased VEGF expression and reduced capillary density in the heart of female mice after pregnancy (21). Treatment of CM-specific PGC-1α KO mice with our E2P4 protocol failed to increase capillary density, indicating a CM-dependent paracrine mechanism. The importance of paracrine secretion of VEGF by CMs for the formation of the vascular network in the heart has been demonstrated in CM-specific VEGF gene deletion mice, which exhibit a severe decrease in capillary density and contractile dysfunction (32). We assume that skeletal muscle cells do not secrete VEGF in response to E2P4 treatment, as capillarization was cardiac-specific and did not increase in the diaphragm or tibialis muscle of the mice. However, the mechanism by which E2 and P4 induce the PGC-1α/ERRα pathway remains unclear, as surprisingly, ER and PR-deficient mice displayed increased capillary density after E2P4 treatment, excluding classical nuclear hormone receptor signaling. These results were further confirmed as no PR expression could be detected in the mouse heart in a transgenic PR-LacZ mouse model. Thus, our experimental results imply a non-canonical regulation of PGC-1α by P4. Such a mechanism has previously been described for PGC-1α via β-adrenergic receptors, whose signal transduction occurs via G-proteins, in skeletal muscle (34). It is therefore possible that in the heart E2 and P4 also signal through G_S_-protein coupled receptors such as GPER or PAQR9 through the cAMP-PKA-CREB pathway. This can be examined in future experiments by measuring changes in the cAMP level in CMs and assessing the phosphorylation status of CREB. *In vivo* the mechanism can be tackled by measuring the angiogenetic effect of E2P4 in CM-specific knockout mouse models for GPER and PAQR9. Furthermore, we cannot exclude an indirect effect of E2P4-action on CMs through another cardiac cell type. E2P4 may stimulate cardiac endothelial cells or fibroblasts to secrete factors that in turn stimulate PGC-1alpha signaling in CMs, leading to the secretion of VEGF.

Taken together, our data reveal how pregnancy induces capillary proliferation specifically in the maternal heart: E2 and P4, elevated during pregnancy, act via the PGC1α/ERRα pathway on CMs to promote VEGF secretion and angiogenesis (Supplementary Figure S5). Surprisingly, this process does not require either the classic P4 or E2 nuclear receptors, suggesting non-canonical signaling by these hormones, as has been reported for macrophages (35). We also show that neither the pregnancy hormones E2 and P4 nor the increased capillarization is sufficient to drive CM hypertrophy, indicating the existence of other signals for the latter, most likely hypervolemia. Our data suggest that targeting this novel pathway in CMs may improve capillarization and blood perfusion without concomitant cardiac hypertrophy in the context of cardiac underperfusion, such as myocardial ischemia.

## Data Availability

The original contributions presented in the study are included in the article/Supplementary Material, further inquiries can be directed to the corresponding authors.
