# Supplementary material for: Pregnancy hormones increase cardiac capillary density via the PGC-1α/ERRα/VEGF pathway in cardiomyocytes
Source: Front Cardiovasc Med. 2025 Oct 14;12:1688831. doi: 10.3389/fcvm.2025.1688831 (PMC12558866; doi:10.3389/fcvm.2025.1688831)
Supplement: Supplementary file 1 [file Datasheet1.docx]

Supplementary Material

# Supplementary Data

# Supplementary Figures and Tables

## Supplementary Figures

**Supplementary Figure 1. Differences in the proliferative rate of cardiac cells during the ovulation cycle; P4 plasma level increase after hormone pellet implantation**

(A) The ovulation cycle in mice is divided into 4 stages: Proestrus, Estrus, Metestrus and Diestrus. The individual stages were determined from vaginal smears that were stained, according to Giemsa. The individual cycles were differentiated by evaluation of the cytological characteristics. (B,C) Cardiac cell proliferation of non-pregnant mice and during different stages of the ovulation cycle was quantified by immunofluorescence staining of heart sections with the cell cycle marker Ki-67. (B) Quantification of non-pregnant mice separated into a highly proliferative group (group 2, G 2) and a weakly proliferative group (group 1, G 1). (C) Analysis of cardiac cell proliferation during the ovulatory cycle showed significantly increased proliferation rates during diestrus. (D) Hormone pellets for continuous hormone delivery over 21 days were implanted into mice 21 days after ovariectomy and mice were analysed after 21 days. P4 plasma levels were quantified by ELISA at the start of the experiment (0=day of pellet implantation), after 1 week (=1), after 2 weeks (=2), and at the end of the experiment after 3 weeks (=3). Data are presented as mean ± SEM.

**Supplementary Figure 2.** **Optimization of hormone treatment regimen to increase capillary density in the heart**

(A) Immunofluorescence staining of capillaries with GSL-I (red) and CM membranes with WGA (green) in heart sections of non-pregnant mice after 14 days of hormone (E2, P4, E2P4) or sesame oil control (CTRL) treatment. Scale bars = 20 μm. (B) Quantitation of capillaries per CM. E2P4: one-factorial ANOVA. n≥4 hearts studied. (C) ELISA analysis of P4 concentration in blood plasma (ng/ml plasma) after 14-day treatment (sesame oil (CTRL), E2, P4, and E2P4). (D) E2 concentration in E2-treated animals (E2, E2P4) and control group (CTRL). n≥4. Data are presented as mean ± SEM. Statistical significance was assessed using a one-way ANOVA with Dunnett’s test for multiple comparison.

**Supplementary Figure 3. E2P4 treatment has no effect on the vascularization of skeletal muscles**

(A-B) Capillaries in histological sections of the tibialis muscle (A) and diaphragm (B) were identified by immunofluorescence staining with GSL-I (red) after 14 days of sesame oil (top) or E2P4 (bottom) treatment. Cell membranes (green) were labeled by co-staining with WGA, and nuclei (blue) were labeled by Hoechst. Scale bars = 50 μm. (C) Quantitation of capillaries per muscle cell in the tibialis muscle and diaphragm of E2P4 treated mice, (n=3). Unpaired t-test (two-sided). Data correspond to mean ± SEM. (D) LacZ-staining of hearts and uterus from PR-AB deficient mice. The PR-AB gene was deleted by knock-in of a lacZ-cDNA into the PR-AB locus, thereby bringing its expression under the control of the endogenous PR-AB promoter. Scale bars = 100 µm.

**Supplementary Figure 4.** **EM-based** **morphological changes in capillaries are consistent with VEGF-A stimulated angiogenesis**

(A-B) Transmission electron micrographs of capillaries in the hearts during gestation (ST 14) and after 14 days of treatment with E2P4 or sesame oil. The images show the morphology of a capillary consisting of ECs (EZ, green arrows), pericytes (white arrows), and the basal lamina (BL, red arrows). The black arrows mark the locations where fenestration of the endothelium is present, which functionally corresponds to an increased capillary permeability. Particularly, the vessel phenotype in (B) suggests an angiogenic activation, as it is observed by enhanced levels of VEGF or VEGF and angiopoietin-2. Scale bars = 1000 nm.

**Supplementary Figure 5.** **Scheme of the proposed mechanism underlying the effect E2P4 treatment**

The application of the steroid hormones E2P4 leads to the expression and/or activation of PGC-1α and ERRα in the heart. The co-activation of ERRα by PGC-1α drives the production and secretion of VEGF by CMs and thereby induces the formation of new vessels in the heart. Capillary density increases without hypertrophy.
